# A pilot study to understand feasibility and acceptability of stool and cord blood sample collection for a large-scale longitudinal birth cohort

**DOI:** 10.1186/s12884-017-1627-7

**Published:** 2017-12-28

**Authors:** S. R. Bailey, C. L. Townsend, H. Dent, C. Mallet, E. Tsaliki, E. M. Riley, M. Noursadeghi, T. D. Lawley, A. J. Rodger, P. Brocklehurst, N. Field

**Affiliations:** 1 UCL Institute of Child Health, University College London, London, UK; 2 University College London Hospital, London, UK; 3 Division of Infection and Immunity, University College London, London, UK; 4 Faculty of Infectious and Tropical Diseases, London School of Hygiene and Tropical Medicine, London, UK; 5 Wellcome Trust Sanger Institute, Cambridge, UK; 6 Institute for Global Health, University College London, London, UK; 7 Institute for Women’s Health, University College London, London, UK; 8 Institute of Applied Health Research, University of Birmingham, Birmingham, UK

**Keywords:** Biological samples, Infant faeces, Cord blood, Feasibility, Acceptability, Large-scale birth cohorts, Bioarchive

## Abstract

**Background:**

Few data are available to guide biological sample collection around the time of birth for large-scale birth cohorts. We are designing a large UK birth cohort to investigate the role of infection and the developing immune system in determining future health and disease. We undertook a pilot to develop methodology for the main study, gain practical experience of collecting samples, and understand the acceptability of sample collection to women in late pregnancy.

**Methods:**

Between February–July 2014, we piloted the feasibility and acceptability of collecting maternal stool, baby stool and cord blood samples from participants recruited at prolonged pregnancy and planned pre-labour caesarean section clinics at University College London Hospital. Participating women were asked to complete acceptability questionnaires.

**Results:**

Overall, 265 women were approached and 171 (65%) participated, with ≥1 sample collected from 113 women or their baby (66%). Women had a mean age of 34 years, were primarily of white ethnicity (130/166, 78%), and half were nulliparous (86/169, 51%). Women undergoing planned pre-labour caesarean section were more likely than those who delivered vaginally to provide ≥1 sample (98% vs 54%), but less likely to provide maternal stool (10% vs 43%). Pre-sample questionnaires were completed by 110/171 women (64%). Most women reported feeling comfortable with samples being collected from their baby (<10% uncomfortable), but were less comfortable about their own stool (19% uncomfortable) or a vaginal swab (24% uncomfortable).

**Conclusions:**

It is possible to collect a range of biological samples from women around the time of delivery, and this was acceptable for most women. These data inform study design and protocol development for large-scale birth cohorts.

**Electronic supplementary material:**

The online version of this article (10.1186/s12884-017-1627-7) contains supplementary material, which is available to authorized users.

## Background

Large-scale cohort studies increasingly collect biological samples which, combined with survey data, might provide valuable insights into the biological mechanisms of disease [1–4]. Storage of samples in large long-term bioarchives has become common, with the aim of using both current and anticipated future molecular technologies to analyse the samples [5, 6]. Reductions in costs of molecular testing, in particular for genomics, proteomics and metabolomics applications, have made such approaches attractive to public health researchers [2, 7]. However, collecting such samples poses multiple challenges, including how to ensure that the proportion of participants providing samples is sufficiently high, and practical challenges associated with working in clinical settings where medical considerations often take priority over research [8, 9].

We are designing a new large-scale UK birth cohort study to investigate how exposure to microorganisms and immunological events during pregnancy and early life influence health and disease outcomes across the life course [10].

We undertook the experimental pilot study described here to: (1) inform decisions about methodology for the main study, (2) develop experience about the practicalities and logistical challenges of collecting samples, and (3) gain understanding of the acceptability of sample collection to women in the late stages of pregnancy and around the time of delivery.

## Methods

### Study design

We aimed to collect samples from approximately 100 women around the time of delivery at University College London Hospital (UCLH). Women were recruited between 17th February 2014 and 4th July 2014 by two research midwives, working normal office hours (9 am to 5 pm). All pregnant women with singleton pregnancies attending either a prolonged pregnancy clinic (gestation 41^+5 or 6^) or a planned pre-labour caesarean section clinic up to a week prior to expected delivery were eligible. We selected these groups as containing women who attend outpatient appointments and who might therefore be approached by study midwives in the week prior to labour to discuss the study. Women with multiple pregnancies, those who needed a translator to consent to participate, those under 16 years of age and non-UK residents who intended to return abroad immediately after delivery were excluded.

We aimed for a sample size of 100 participants to estimate with reasonable precision the proportion returning each of the sample types. The study was not designed or powered to detect differences by subgroups.

### Sample collection

Cord blood and stool were chosen for collection as these samples were subject to the greatest uncertainty regarding the optimum processes required for collection, as well as acceptability to women. Study protocols developed as a result of this pilot study are available in Additional file 1.

Cord blood was collected in the maternity unit soon after delivery by a research midwife or by the attending midwife if the delivery occurred outside normal office hours. Cord blood banking was available at UCLH for those women who wanted to donate cord blood and this service was given precedence where women agreed to both.

Women were asked to provide a stool sample in the maternity unit before or after delivery, or stool was collected during delivery by midwives. The first baby stool sample (meconium) was collected while the baby remained in hospital. For a subset of ten women recruited in May and June 2014, an additional baby stool collection protocol was established and tested for home collection of stool every other day after birth: samples were taken by women and collected from their home by courier. These samples were used for experimental validation of our procedures. After home collection of baby stool commenced, women not enrolled in home collection were only asked to consent to collection of cord blood and maternal stool. Home collection of baby stool ended on 9th June 2014, after which all collection of maternal stool and baby stool collection stopped; only cord blood was collected from this date until the end of the study (4th July 2014). We were not able to collect reasons for failing to get a sample.

### Questionnaires

Women were asked to self-complete paper-based questionnaires before (all women) and after (only women who provided samples) sample collection to assess the acceptability of collecting samples in this pilot as well as the acceptability of additional samples planned for the main study (maternal and baby urine, vaginal swabs, placental tissue and baby saliva). The pre-sample and post-sample questionnaires are available in Additional files 2 and 3, respectively.

Women were asked for their opinions about and satisfaction with the study overall as well as about the acceptability of providing each sample using a five point Likert scale (“very satisfied / comfortable” to “very unsatisfied / uncomfortable”). Women could also provide free-text comments.

### Data analysis

Comparisons were made between women who did and not provide at least one sample. Comparisons were also made by sample type for women who provided at least one sample. For maternal stool, the denominator for these analyses included only those women who were approached to provide a stool sample (*n* = 85).

Comparisons of continuous variables, such as maternal age, were made using Student’s t-tests. Categorical variables were compared using either Chi-squared or Fisher’s exact tests. Means across groups were compared using one-way analysis of variance (ANOVA). All analyses were carried out using STATA version 12.0.

## Results

**Fig. 1:**
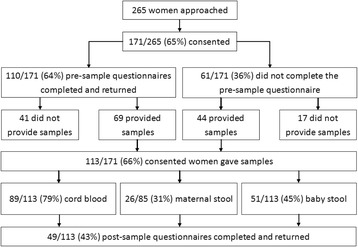
Study recruitment and sample collection

Of 265 women approached, 171 (65%) of women consented to participate and 113 (66%) of these women gave at least one sample (Fig. 1). Seventeen women consented to participate but did not provide any samples or complete a pre-sample questionnaire.

The mean age of women who consented to participate was 34 years. Women were primarily of white ethnicity (130/166, 78%) and half were nulliparous (86/169, 51%) (Table 1). Among 168 women where mode of delivery was recorded, 57% delivered vaginally (unassisted or instrumental), nearly one third (29%) by planned pre-labour caesarean section, and 15% by intrapartum caesarean section. For 140 women who consented to participate and where information on day and time of birth were recorded, approximately half delivered outside of normal office hours (75/140, 54%).

**Table 1:** Demographic characteristics of women who consented to participate, and the proportion providing ≥1 sample

	All		Sample given		*P*-value
Characteristic	n	%	n	%	
Woman’s ethnicity^a^					
White	130	78	85	65	
Asian	12	7	8	67	
Black	14	8	11	79	
Mixed/Chinese/other	10	6	5	50	
Total	166	–	109	–	0.56
Parity^a^					
0	86	51	57	66	
1	63	37	42	67	
>1	20	12	14	70	
Total	169	–		–	1.00
Mode of delivery^a^					
Vaginal	95	57	51	54	
Unassisted	70	42	35	50	
Forceps	15	9	11	73	
Ventouse	10	6	5	50	
Planned pre-labour caesarean	48	29	47	98	
Intrapartum caesarean	25	15	15	60	
Total	168	–	113	–	< 0.001
Woman given antibiotics at any time^a^					
Yes	68		59	87	
No	39		33	85	
Total	107		92	–	0.76
Infant given antibiotics^a^					
Yes	12		11	92	
No	117		91	78	
Total	129		102	–	0.26

^a^In participants where data were available

Women who delivered by planned pre-labour caesarean section were most likely to provide at least one sample (98%) and those delivering by unassisted spontaneous vaginal (50%) or ventouse were least likely (50%) to provide a sample (Table 1). In 110 women who provided at least one sample and where information on day and time of birth were recorded, 47% (52/110) delivered outside of normal office hours. We observed no significant difference between whether a sample was given and maternal age (*p* = 0.09, continuous variable), ethnicity (*p* = 0.56) or parity (*p* = 1.00), although we note the relatively small sample size in this pilot study, which may have been underpowered to detect any difference.

Of the 113 women from whom at least one sample was collected, maternal stool was the least frequently collected sample (26/85, 31%), while cord blood collection was most frequently collected (89/113, 78%). Midwives stopped collecting baby meconium after 6th May 2014 because this sample was found to contain no bacterial nucleic acid signature. Up to this date, 45 out of 70 women (64%) had provided a meconium sample from their baby.

Whether a maternal stool sample was collected was associated with delivery mode (*p* = 0.01) (Table 2). Maternal stool samples were more likely to be given by women who delivered by spontaneous vaginal delivery (14/29) and least likely to be given by women who had planned pre-labour caesarean sections (3/31) (Table 2).

**Table 2:** Association between demographic characteristics and sample collection for women who provided ≥1 sample

Characteristic	Total who gave any sample	Cord blood given		*P*-value	Total asked to provide any stool sample^b^	Maternal stool given		*P*-value
		*n*	%			*n*	%	
Woman’s ethnicity^a^								
White	85	66	78		65	21		
Asian	8	5	63		5	2		
Black	11	10	91		8	1		
Mixed/ Chinese/other	5	5	100		4	1		
Total	109	86	–	0.37	84	25	–	0.77
Parity^a^								
0	57	44	77		45	13		
1	42	32	76		32	11		
>1	14	13	93		9	2		
Total	113	89	–	0.47	85	26	–	0.74
Mode of delivery^a^								
Vaginal								
Unassisted	35	25	71		29	14	48	
Forceps	11	7	64		9	4	44	
Ventouse	5	3	60		5	1	20	
Planned pre-labour caesarean	47	42	89		31	3	10	
Intrapartum caesarean	15	12	80		11	4	36	
Total	113	89	–	0.09	85	26	–	0.01

^a^In participants where data were available

^b^A total of 85 women were asked to provide maternal stool

We observed no evidence of an association between collection of cord blood and any of the demographic characteristics measured (Table 2). The overall mean volume of blood collected was 12.6 ml (range: 1 to 40 ml), and this was not associated with either mode of delivery (*p* = 0.10: one way ANOVA).

One hundred and ten of 171 (64%) women who agreed to take part in the study completed a pre-sample questionnaire, including 41 women who did not provide a sample (Fig. 1). Over 95% of women reported that they were satisfied with the explanation about the study, why samples needed to be collected, and what taking part involved (105/110, 95%; 108/110, 98%; and 110/110, 100%, respectively). Women generally felt comfortable about samples being collected from their baby (Table 3). Most women (67%) also reported being comfortable or very comfortable with giving their own stool sample (Table 3). Fewer women felt comfortable about giving a vaginal swab (53%) (Table 3).

**Table 3:** Pre- and post-sample responses to the question, ‘Overall, how comfortable do you feel with the idea of providing the following samples?’

	Pre-sample		Post-sample	
Sample	*n*	%	*n*	%
Maternal stool				
Very comfortable / comfortable	72	67	27	57
Neither	16	15	3	6
Uncomfortable / very uncomfortable	19	18	17	36
Total	107		47	
Maternal urine				
Very comfortable / comfortable	96	97	41	93
Neither	2	2	2	5
Uncomfortable/Very uncomfortable	1	1	1	2
Total	99		44	
Maternal vaginal swab				
Very comfortable / comfortable	52	53	26	59
Neither	22	22	7	16
Uncomfortable / very uncomfortable	24	25	11	25
Total	98		44	
Cord blood				
Very comfortable / comfortable	97	87	47	96
Neither	7	7	2	4
Uncomfortable / very uncomfortable	7	7	0	0
Total	108		49	
Stool from baby’s nappy				
Very comfortable / comfortable	100	93	43	92
Neither	3	3	3	6
Uncomfortable / very uncomfortable	5	5	1	2
Total	108		47	
Urine from baby’s nappy				
Very comfortable / comfortable	91	93	–	–
Neither	4	4	–	–
Uncomfortable / very uncomfortable	3	3	–	–
Total	98		–	–
Saliva from baby’s mouth				
Very comfortable / comfortable	82	84	–	–
Neither	7	7	–	–
Uncomfortable / very uncomfortable	9	9	–	–
Total	98		–	–

34 women responded to both questionnaires

Forty-nine of the 113 (43%) women who gave at least one sample returned a post-sample questionnaire. Overall satisfaction with study explanations was very similar to the pre-sample questionnaires (data not shown), and most women reported that they were comfortable with sample collection in the post-sample questionnaire (Table 3).

Free text feedback was provided by 64 women. Several women reported feeling uncomfortable about the idea of having a vaginal swab collected, which they felt might be invasive and painful. Eleven women (17%) reported feeling uncomfortable about providing their own stool due to embarrassment and because this is not a routine procedure (in contrast to collection of urine). Seven women (11%) raised concerns about posting baby stool samples due to the demands of caring for a newborn baby.

## Discussion

This study provides evidence about biological sample collection rates from women in late pregnancy, at around the time of delivery and from their babies, and provides insight into whether or not women feel comfortable with sample collection. Overall, sample collection success was good, with 66% (113/171) of women recruited providing at least one sample and most reporting that they found the study acceptable.

In general, samples were more likely to be provided when delivery was by planned pre-labour caesarean section (except maternal stool), probably because pre-labour caesarean sections were easier to plan and prepare for, as the time of delivery was known in advance. The Monday to Friday working pattern of the study’s research midwives may also have impacted on sample collection success; 54% of women who consented to participate delivered outside of normal office hours. A major constraint on sample collection may therefore have been this practical issue rather than a lack of willingness from women having vaginal deliveries. This is reflected in both the percent of those approached who consented to participate (171/265, 65%) and in generally high levels of acceptability for most samples in responses to pre-sample questionnaires.

The collection rate for maternal stool samples was low at 31%. This may have been due in part to our deliberate selection of women who delivered by planned pre-labour caesarean section, from whom fewer stool samples were collected, probably because passing of stool during C-section delivery is unlikely. Since fewer deliveries would be by caesarean section in a population-based study [9], we anticipate that maternal stool sample collection may be higher in our main study.

It was encouraging that the proportion of women from whom cord blood was collected was high (79%), despite the fact that cord blood banking was given precedence over our own sample collection. In the absence of cord blood banking, samples could potentially be collected from a higher number of participants, and/or larger volumes obtained.

Overall, the study and procedures were acceptable to most participants, with particularly high acceptability of collection of maternal and baby urine, baby stool and cord blood. We were surprised that the collection of vaginal swabs was perceived to be painful to collect or too invasive because such swabs have been shown to be highly acceptable in other settings such as sexual health clinics and for cervical smear screening [11, 12]. Such concerns may be alleviated by enabling self-collection and providing more information about what the procedures involve.

A limitation of this study is that it was carried out in specific late pregnancy clinics in a central London teaching hospital, and the women recruited might therefore not be representative of pregnant women in the general population. Moreover, some selection bias is possible, with those women who participated being more likely to find the study acceptable. For a small number of participants, some data items were missing. Due to the scale of this pilot we were not able to include women who needed a translator to understand the study materials. For women who consented to take part but did not provide a samples, we were unable to determine whether this was because their changed their minds or because they lacked an opportunity to provide the sample.

Our pilot found similar or somewhat better cord blood collection rates than other studies. For example, a pilot study for the ELFE longitudinal cohort in France reported cord blood collection for 82% of participating mothers, which was similar to our study, [13] whereas a feasibility study in Germany reported cord blood collection for 54% of women who consented [6]. Few gut microbiota studies report response rates or information about acceptability of stool collection, and most to date have been small in scale [14, 15]. Where reported, stool sample response rates have varied considerably between studies. For example, the Canadian CHILD study reported 3-month and 1-year stool samples collected at home by visiting nurses were available for 33% (422/1264) babies, [16] while stool samples collected from children aged 1–11 months were available for 24% of participants in the American WHEAL cohort study, [17] and a feasibility study in Germany in 75 1- to 3-year-old children reported stool sample for 65.3% of participants [9]. Overall, we found few examples in the literature of dedicated studies describing the acceptability, responses rates and challenges when collecting cord blood and stool from mothers and babies around the time of birth, which emphasises the importance of the data reported here.

## Conclusions

Overall, our study indicates that it is acceptable and possible to collect biological samples from pregnant women in a clinical setting using research midwives and routine National Health Service (NHS) staff, which has important implications for study design and protocol development for large-scale birth cohorts collecting biological samples around the time of birth. The major constraints to sample collection are likely to be logistical rather than related to acceptability and willingness to participate.

## Additional files

Additional file 1: Baby Biome Study Collection and processing protocol: samples at birth. (PDF 2102 kb)

Additional file 2: Pre-birth questionnaire, pilot study questionnaire given to all women who consented to participate prior to sample collection. (PDF 368 kb)

Additional file 3: Post-birth questionnaire, pilot study questionnaire given to all women who gave at least one sample. (PDF 357 kb)

## Abbreviations

ANOVA: Analysis of variance
NHS: National Health Service
UCLH: University College London Hospital
