# Supplementary material for: A pilot study to understand feasibility and acceptability of stool and cord blood sample collection for a large-scale longitudinal birth cohort
Source: BMC Pregnancy Childbirth. 2017 Dec 28;17:439. doi: 10.1186/s12884-017-1627-7 (PMC5745976; doi:10.1186/s12884-017-1627-7)
Supplement: Supplementary file 1 — Baby Biome Study Collection and processing protocol: samples at birth. (PDF 2102 kb) [file 12884_2017_1627_MOESM1_ESM.pdf]

THE BABY BIOME STUDY

Collection and processing protocol: samples at birth

Contents

1. Introduction ... 4
1.1. The purpose of this document ... 4
1.2. Background ... 4
1.3. Baby Biome Study samples ... 4
1.4. Information system ... 4
2. General information ... 6
2.1. Consent ... 6
2.2. Inclusion criteria ... 6
2.3. Exclusion criteria ... 6
2.4. Identification of participants ... 6
2.5. Feedback of results from biological samples ... 6
Section A: Acknowledgements ... 7
1. Baby Biome Study team ... 7
2. BHRUT staff ... 7
3. University Hospitals Leicester (UHL) staff ... 7
4. Other contributors ... 7
Section B: Collection of samples ... 8
1. General points ... 8
1.1. Labelling of samples ... 8
1.2. Consent forms ... 8
1.3. Data collection form and midwife checklist ... 9
1.4. Log book ... 9
1.5. Transfer of samples to the laboratory ... 9
2. Sample-specific collection protocols ... 9
2.1. Vaginal swab ... Error! Bookmark not defined.
2.2. Maternal stool ... Error! Bookmark not defined.
2.3. Cord blood ... 17
2.4. Baby stool collected at home ... Error! Bookmark not defined.
Section C: Processing and aliquoting of samples ... 24
1. General specimen processing ... 24
1.1. Specimen handling, disposal and work bench clean up ... 24
1.2. Specimen labelling ... 24

1.3. General equipment/materials ... 25
1.4. Handling of contaminated samples, incidents and accidents ... 25
1.5. Reporting of contaminated samples ... 26
2. Sample specific processing ... 27
2.1. Vaginal swab ... 27
2.2. Maternal stool ... 28
2.3. Cord blood – Serum ... 31
2.4. Cord blood – RNA ... 32
2.5. Cord blood – stimulation tube ... 32
2.6. Baby stool ... 33
Section D: Shipment of samples to external sites ... 37
1. Data and sample preparation ... 37
1.1 Shipment Information Form (data preparation) ... 37
1.2 Sample preparation ... 37
Section E: Appendices ... 39
1a. Sample Auditing Log ... 39
1b. Incidents and Accidents Log ... 41

1. Introduction

1.1. The purpose of this document

This document describes the standard operating procedures (SOPs) for collecting and processing biological samples for the Baby Biome Study.

1.2. Background

The Baby Biome Study is a birth cohort designed to recruit up to 40,000 children across multiple hospital sites in England. The Baby Biome Study has a specific focus on how interactions between microorganisms, both pathogenic and the colonising microbiota, the immune system and clinical, social and behavioural factors during pregnancy, birth, infancy and childhood influence health throughout life.

Women will be approached during pregnancy with information about the Baby Biome Study. When women arrive at the labour ward to give birth, they will then be approached again to provide a set of samples (vaginal swab, maternal stool, cord blood and baby stool) and to consent to participation.

Section A of this document provides SOPs for collection of samples and Section B provides SOPs for processing and storage of samples.

1.3. Baby Biome Study samples

Table 1 summarises the biological samples that will be collected for the Baby Biome Study around the time of birth.

Table 1 **Summary of samples for the Baby Biome Study**

		Vaginal swab	Maternal stool	Baby stool			Cord blood		
				4 days	7 days	21 days	RNA	Serum	ex vivo
Birth	Collected?	x	x	x			x	x	x
	Location of collection	Labour ward		Participant's home			Labour ward		
	Collected by	Participant	Labour ward midwife or participant	Participant			Labour ward midwife		
	Processing location	Pathology laboratory at Queens Hospital							
	Processed by	MLA 1 & 2							

1.4. Information system

Barcoding

Upon receipt in the pathology laboratory at BHRUT, samples will be processed and aliquoted into pre-barcoded cryogenic vials for anonymisation purposes and a study ID will be generated. Barcodes will be linked to participant and sample data in the study database using a barcode scanner.

IT and Database

A Customer Relationship Management Tool (CRM) has been developed for the Baby Biome Study for electronic data collection. The system will hold records for each participant, including information about their biological samples. Tables 2a and b show the data that will be recorded in relation to these samples.

Table 2a **Example general collection and processing metadata**

Field	Data Type
Laboratory System ID	Text (pre-populated by the system)
Sample collected or not (with reason for not collecting if known)	Boolean checkbox (with free-text field for other)
Time of sample collection from participant	Date and Time
Sample volume / amount	Whole Number + unit of measure
Time the sample arrives to labour ward sluice room	Date and time
Time sample is processed/aliquoted	Date and Time
Actual number of aliquots stored	Whole Number
Volume / weight of each aliquot	Whole number + unit of measure ml
Date and time to freezer	Date and Time
Sample error field (inadequate vol, wrong tube, not labelled, spillage, contamination, lost sample. .other)	Drop-Down (with free-text field for other)
Time aliquot is stored at ultralow temperature at biorepository	Date and time
Free text field	Text

Table 2b **Example sample specific data**

Field	Data Type
Serum haemolysis	Boolean checkbox
Type of stool sample	Drop-down (DNA extraction or raw sample)
Maternal stool DNA concentration	0.000

Infant stool DNA concentration	0.000
Lot number of the DNA extraction kit	Whole Number
Code number for negative control	Whole Number
Date and time to fridge for stool	Date and Time

2. General information

2.1. Consent

Women should be asked to provide consent after birth whilst on the labour ward, by their attending midwife. Some women may wish to consent before birth. Some women, if they require more time to decide, may be asked to consent whilst on the postnatal ward.

2.2. Inclusion criteria

All pregnant women who arrive at the labour ward to give birth.

2.3. Exclusion criteria

The following women will be excluded from taking part:

- Women under 16 years of age
- Non-UK residents who intend to return abroad immediately after delivery

2.4. Identification of participants

Any woman who arrives at the labour ward to give birth may be approached to participate by giving them a Participant Information Sheet (PIS). Women who have declined participation during pregnancy will have a sticker placed on the front of their medical notes indicating that they should not be approached again when they arrive at the hospital to give birth.

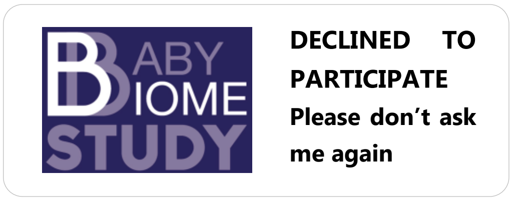

Figure 1 **Sticker indicating participation declined**

2.5. Feedback of results from biological samples

There will be no feedback of results for participants from any tests undertaken on the samples described in this document. The tests we perform will not be used for diagnostic or clinical purposes and the results will take at least several months to become available and in most cases much longer. As such, we won't be able to return any results for the tests performed to individual participants.

Section A: Acknowledgements

1. Baby Biome Study team

The SOPs in this document were developed by the Baby Biome Study core team comprising **Prof Peter Brocklehurst**, **Dr Nigel Field**, **Dr Alison Rodger**, **Dr Claire Townsend**, **Ms Sarah Bailey**, **Dr Helga Laszlo** and **Mr Byron Brown**.

Research midwives **Camille Mallet** and **Helen Dent** developed collection protocols for cord blood, maternal and baby stool samples and piloted these protocols during development work carried out at UCLH.

Research midwives **Bernie Ahern** and **Zoe Turoff** provided input to refine collection protocols for all samples according to practice at Barking, Havering and Redbridge University NHS Trust.

Dr Evi Tsaliki developed collection protocols for cord blood, maternal and baby stool samples and undertook experimental work to develop and finalise processing protocols.

Dr Mahdad Noursadeghi advised on the development of collection and processing protocols for cord blood serum and RNA and undertook experimental work to inform the RNA processing protocol.

Professor Eleanor Riley (London School of Hygiene and Tropical Medicine) informed the development of cord blood collection and processing protocols.

Dr Trevor Lawley (Sanger Institute, Cambridge) provided a protocol for baby stool collection, informed the development of processing protocols for maternal and baby stool and undertook experimental work to determine the optimum time after birth to collect baby stool samples.

2. BHRUT staff

Rob Davis and **Cherian Koshy** from the Pathology department at BHRUT advised on the protocols to ensure they meet NHS and local hospital standards.

3. University Hospitals Leicester (UHL) staff

Research support officer **Sarah Clarke** provided input to refine collection and processing protocols for all samples according to practice at University Hospitals Leicester NHS Trust.

4. Other contributors

The following contributors provided SOPs that formed the basis of SOPs developed for the Baby Biome Study:

> **Dr Jacques Ravel** (University of Maryland) provided the protocol for vaginal swab self-collection and advised on the vaginal swab processing protocol.

The following contributors provided further information which informed the SOPs in this document:

> **Dr Nitin Kumar** (Sanger Institute, Cambridge) contributed towards the stool samples' collection, handling, processing and storage protocols.
>
> **Dr Mark Stares** (Sanger Institute, Cambridge) contributed towards the stool samples' collection, handling, processing and storage protocols.

Section B: Collection of samples

1. General points

Midwives will be expected to collect or assist women in collecting all the samples where necessary. Sample collection kits can be found on the labour ward in delivery rooms and in the labour ward sluice room.

1.1. Labelling of samples

Samples should be labelled by attaching one of the woman's hospital stickers to the designated space on a blank Baby Biome Study label (Figure 2).

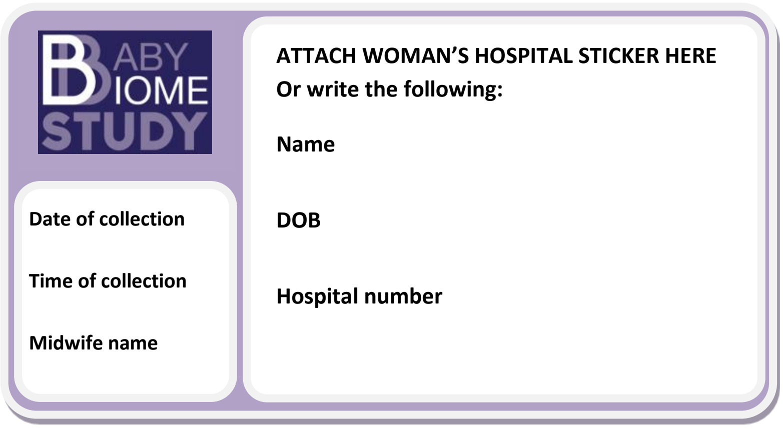

Figure 2 **Baby Biome Study label**

Hospital stickers should contain the following information:

- Name of patient (eye readable)
- Hospital number (eye readable)
- Date of birth (eye readable)

When labelling samples it is important that the date and time of collection are written onto the Baby Biome Study sticker so that the study laboratory technicians know the time between collection and the sample arriving at the laboratory.

The name of the midwife who collected the sample should also be recorded on the Baby Biome Study label.

1.2. Consent forms

Women will need to provide **written consent** to donate their samples to the Baby Biome Study. Consent is to be taken by their attending midwife whilst on the labour ward, after birth. Some women may be happy to consent before birth.

Consent forms can be found in the labour ward sluice room.

The consent form should be labelled with the woman's hospital label and/or a handwritten Baby Biome Study label. One label should be printed for each page of the form (a total of **three** labels).

1.3. Data collection form and midwife checklist

To help the Baby Biome Study answer specific scientific questions about infection and immunity, a data collection form should be completed for all participants. This paper form records basic clinical information like date and time of birth, mode of delivery and birth weight, as well as information about maternal and infant perinatal antibiotic administration. The form should be kept in the woman's maternity notes until it has been completed, prior to discharge from the labour ward. Once completed the form should be placed into the locked Baby Biome Study box in the labour ward sluice room as it contains confidential information.

To ensure that samples are collected from as many women as possible, midwives should also complete the midwife checklist located on page four of the data collection form.

Blank copies of the data collection form / checklist are available in the labour ward sluice room. Ensure that the woman's hospital label is placed onto the front of the data collection form.

1.4. Log book

There is a log book located in the labour ward sluice room. This should be used to record details such as the time samples are placed into the Baby Biome Study fridge or Baby Biome Study box. It is very important that this log is completed correctly as it will provide a way to trace samples. The Baby Biome Study research midwives will maintain a file of completed log book sheets.

1.5. Transfer of samples to the laboratory

All samples should be taken to the sluice room on the labour ward immediately after collection by midwives. Samples will need to be placed either into the Baby Biome Study fridge or into a box at room temperature.

The Baby Biome Study laboratory technician will be responsible for transferring samples to the pathology laboratory twice daily where they will be kept at the appropriate temperature and once a day at weekends. If the labour ward fridge becomes full midwives should call the laboratory and ask the Baby Biome Study technician to transfer the samples. At weekends and on Bank Holidays a technician may be contacted to collect samples if the Baby Biome Study fridge becomes full.

2. Sample-specific collection protocols

2.1. Vaginal swab

Equipment

a. Collection kit for women:

COPAN 480C Elution swab and collection tube containing 1ml liquid Amies (red cap)

Cup/holder for collection tube
Self-collection instruction sheet

b. Additional equipment:

Non-sterile gloves
1 x Baby Biome Study label
1 x woman's hospital sticker

Timing of sample collection

The sample should be self-collected by the woman during labour, whilst on the labour ward. Alternatively, the woman may prefer the midwife to collect the sample, which should be done at the time of the first digital vaginal examination. The sample can be collected before or after the woman's waters have broken.

Procedure

If the woman has asked her midwife to collect the sample, explain fully what the collection procedure entails and the reasons behind why the sample is being taken. It is worth emphasising that the collection of the sample does not require a speculum examination and can be quickly and easily self-collected without causing any harm or pain. If the woman declines to give the sample the other Baby Biome Study samples may still be collected.

Only use the swabs provided, do NOT substitute with other sponges or swabs.

a. Instructions for women:

Read the following instructions carefully before proceeding to collect your sample.

1. Remove the lid from the collection tube and place the tube upright in the cup provided. Place the cap upside down on a flat surface.
   NOTE: There is liquid in the tube so be careful not to spill it.

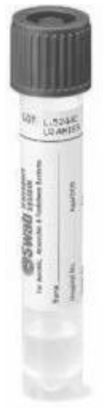

2. Remove the COPAN collection swab from the wrapper, handle end first. Be very careful not to touch the tip of the swab.
   NOTE: Do not touch the swab below the red line.
3. Using your non-dominant hand (not your writing hand) open the labia (lips of the vagina) to allow entrance of the swab into the vagina.
4. Insert the swab two inches into the vagina being careful not to touch the tip of the swab anywhere else in your genital area.

5. Twist the swab three times while inside the vagina. The swab will become saturated with vaginal fluid.
6. Remove the swab the same way that you did for insertion. Again, be careful not to touch the tip of the swab outside of the vagina.
7. Place the swab into the liquid in the collection tube.
8. Gently break the swab at the red line by bending the handle until it snaps. Replace the cap.
   NOTE: Be careful not to spill the liquid in the bottom of the tube.
   NOTE: Ensure that the swab handle is positioned in the centre of the cap so that the cap can be closed easily.

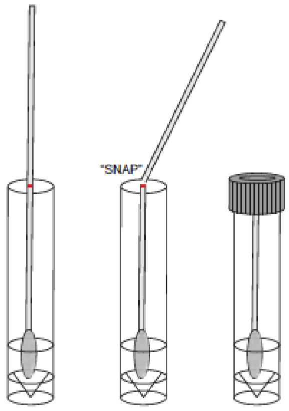

9. Screw the top firmly on the collection tube. Gently swirl the collection tube several times to ensure that the sample mixes with the liquid in the tube.
10. **Ensure that the sample stays in the upright (standing) position** by placing the tube into the cup provided.
11. Give your sample to the attending midwife.

b. Instructions for midwives:

1. Attach the woman's hospital sticker to the Baby Biome Study label.
2. Write the date and time that the sample was collected in the spaces provided.
3. Attach the Baby Biome Study label to the screw top specimen collection tube.
4. Give the collection kit, including the self-collection instructions, swab and specimen tube and a cup/holder for the collected sample, to the woman.
5. Once the woman has returned her sample:
   a. Place the collection tube into the Baby Biome Study fridge in the delivery suite store room. Ensure that the sample is placed vertically into the tube rack in the fridge.
      NOTE: Vaginal samples must be stored at 4°C until they are transferred to the laboratory and **must be kept upright**.
   b. Record whether a sample was given using the Baby Biome Study checklist kept in the woman's notes. If a sample was not given ensure that a reason (e.g. consent declined) is provided.
   c. Complete sample log on the Baby Biome Study fridge.

Vaginal swab self-collection instructions

This is a sample that is very easy to collect yourself if you choose. You should collect your sample during labour, before you give birth. This can be either before or after your waters have broken. Taking the sample will not pose any risk to your baby.

Alternatively, you may prefer the midwife to collect your sample. This might be done during labour before a routine vaginal examination.

If you would like to take the sample yourself, you will be given a collection kit by your midwife during labour. Please only use the swabs provided. Do NOT substitute with other sponges or swabs.

Read the following instructions carefully before you collect your sample.

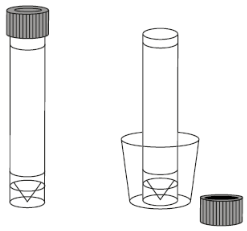

STEP 1
Remove the lid from the collection tube and place the tube upright in the cup provided. Place the lid from the tube upside down on a flat surface.

NOTE: There is liquid in the tube so be careful not to spill it.

STEP 2
Remove the collection swab from the wrapper handle end first. Be very careful not to touch the tip of the swab.

NOTE: Do not touch the swab below the red line.

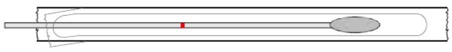

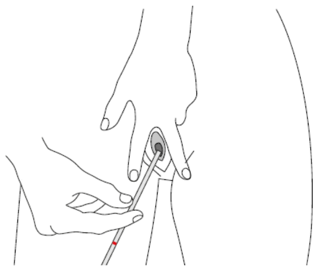

STEP 3
Using your non-dominant hand (not your writing hand) open the labia (lips of the vagina) to allow entrance of the swab into the vagina.

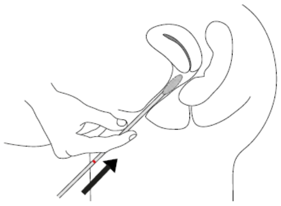

STEP 4
Insert the swab two inches into the vagina being careful not to touch the tip of the swab anywhere else in your genital area.

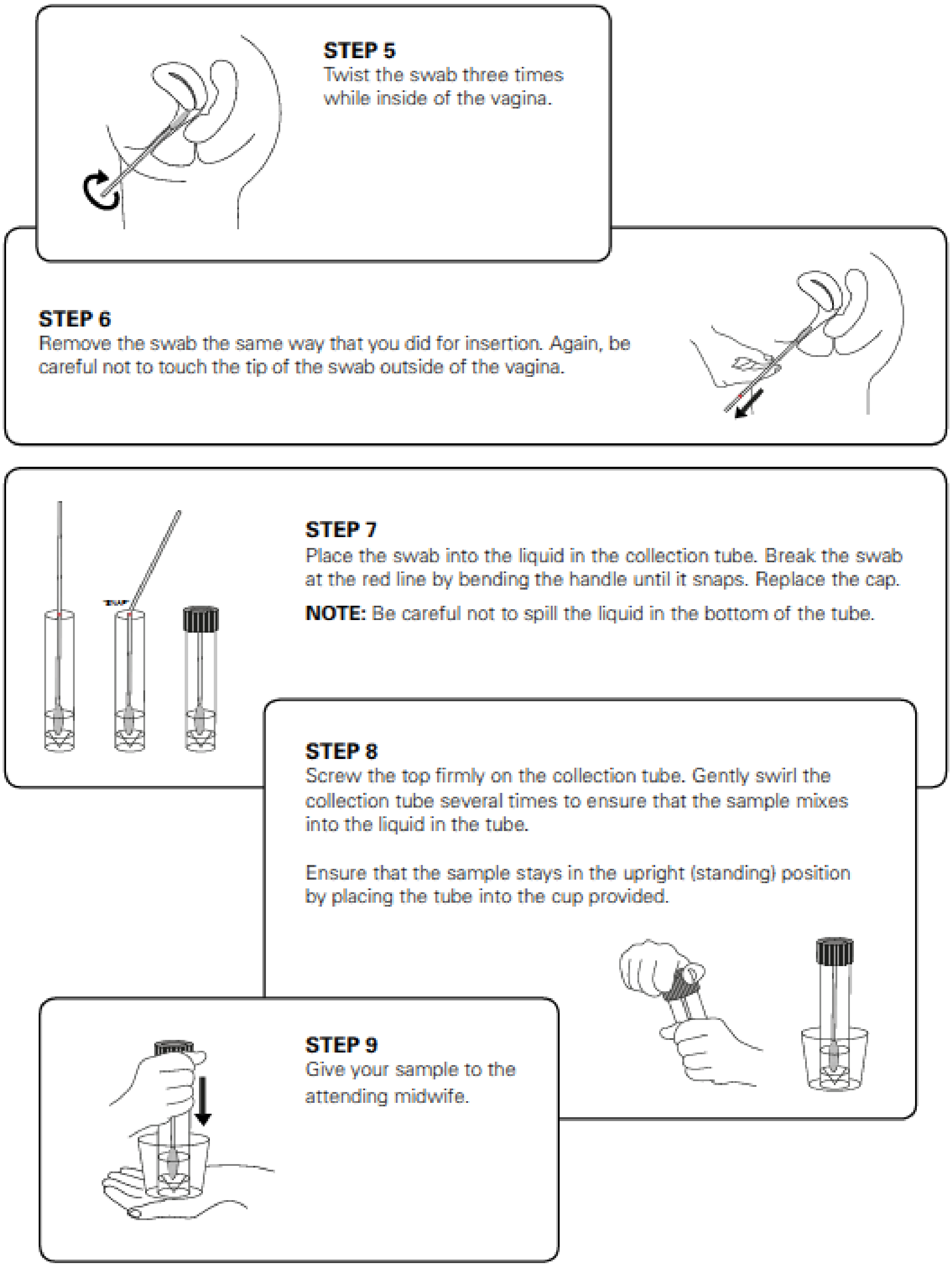

Figure 3 **Steps for self-collection of a vaginal swab**

2.2. Maternal stool

Equipment

a. Collection kit for women:

Stool specimen collection kit (02-544-208, Fisher scientific), which includes a stool specimen frame that sits across the toilet bowl and supports a collection pot

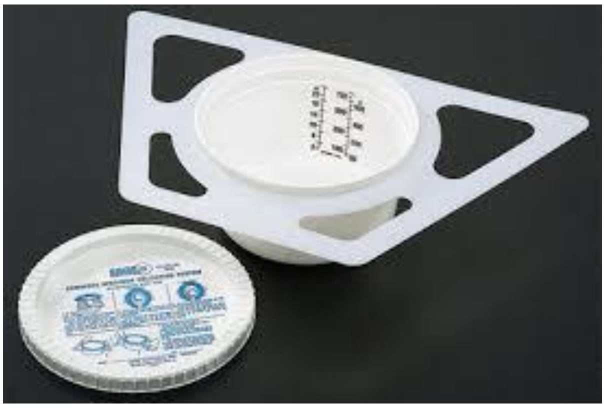

Ziploc plastic bag large enough to hold the specimen collection kit
Sample collection instruction sheet

b. Additional equipment:

Non-sterile gloves
1 x Baby Biome Study label
1 x woman's hospital sticker

Timing of sample collection

Samples are to be collected during the hospital stay ideally during delivery by the attending midwife on the labour ward. Some women may be happy to obtain their own sample before birth, although after the birth is also acceptable.

Procedure

The woman may decline to give the sample but continue to provide the other samples requested.

In many cases, women will pass stool during delivery. The delivering midwife should place the stool passed during delivery directly into one of the stool pots. The sample can be wrapped in an inco sheet or paper when placed into the collection pot.

NOTE: Stool samples **should not** be collected from birthing pools.

Some women may be happy to obtain their own stool sample, either before or after birth, depending on whether stool was passed during delivery. The woman will be provided with a stool specimen collection kit, which includes a stool specimen frame that sits within the toilet and supports a collection pot.

NOTE: Women should try not to urinate into the collection pot.

a. Instructions for midwives (for collection during delivery):

1. When stool is passed during the second stage, place the stool into the specimen collection pot.

   NOTE: The sample can be wrapped in an inco sheet or paper when placed into the collection pot.
2. Place the lid onto the pot and seal it.
3. Attach the woman's hospital sticker to the Baby Biome Study label.
4. Write the date and time that the sample was collected in the spaces provided.
5. Attach the Baby Biome Study label to the specimen collection pot.
6. Take the labelled collection pot containing the sample into the delivery suite store room, place it into a Ziploc bag and put it into the Baby Biome Study fridge.

   NOTE: Stool samples must be stored at 4°C until they are transferred to the laboratory.
7. Wash your hands thoroughly.
8. Record whether a sample was given and whether verbal consent was obtained using the Baby Biome Study checklist kept in the woman's notes. If a sample was not given ensure that a reason (e.g. consent declined) is provided.
9. Complete sample log on the Baby Biome Study fridge.

b. Instructions for midwives (for self-collection by woman):

1. Give the collection kit, including the stool collection pot, the Ziploc bag and sample collection instructions (see Figure 4 below), to the woman.
2. Once the sample has been placed into the pot the woman will return the sample to her attending midwife.

c. Instructions for midwives (once a sample has been collected):

3. Put on clean non-sterile gloves.
4. Take the collection pot containing the sample into the delivery suite store room.
5. Remove the collection pot from the Ziploc bag and ensure that the lid is firmly clipped onto the collection pot (lid should snap into place).
6. Attach the woman's hospital sticker to the Baby Biome Study label.
7. Write the date and time that the sample was collected in the spaces provided.
8. Attach the Baby Biome Study label to the specimen collection pot.
9. Place the stool collection pot containing the sample back into the Ziploc bag and place it into the Baby Biome Study fridge in the delivery suite store room.

   NOTE: Stool samples must be stored at 4°C until they are transferred to the laboratory.
10. Dispose of the gloves.
11. Wash your hands thoroughly.
12. Record whether a sample was given using the Baby Biome Study checklist kept in the woman's notes. If a sample was not given ensure that a reason (e.g. consent declined) is provided.
13. Complete sample log on the Baby Biome Study fridge.

Stool collection instructions for adults

You have been provided with a poo (stool) collection kit that includes a collection pot and frame as well as a Ziploc bag to put the pot into once the sample is collected.

Please discuss with your midwife if you have any questions or concerns.

STEP 1
Raise the toilet seat. Place the poo collection frame on the back of the toilet bowl. All four corners of the collection frame should be supported by the toilet bowl. Place collection bowl in frame. Place toilet seat down.

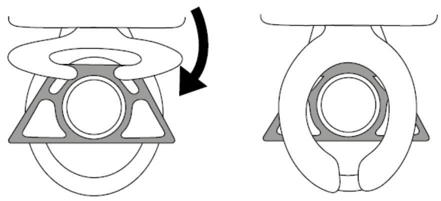

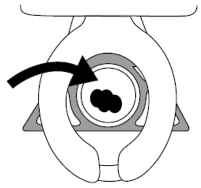

STEP 2
Deposit your poo directly into the collection container. Please try not to urinate into the collection container.

(If accidental urination occurs, we will still be able to use the sample but it would be very helpful if this could be avoided.)

STEP 3
After collecting your specimen, remove the container from the frame. Place the container on a flat surface and firmly press the lid closed.

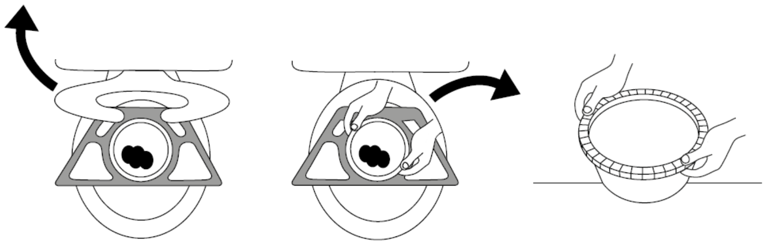

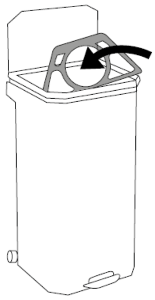

STEP 4
Discard collection frame in hospital waste.

STEP 5
Place the closed container into the Ziploc bag, seal the bag and return the sample to your midwife who will make sure the pot is properly labelled.

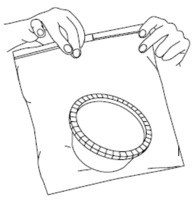

Figure 4 **Steps for self-collection of maternal stool**

2.3. Cord blood

Equipment

a. Collection kit:

2 x cream butterfly needles (size 19G) with **no** pre-attached vacutainer holder
1 x 20ml syringes
Blood bottles:

- Tempus™ Blood RNA Tube (blue cap) (Life Technologies, 4342792)
- Serum clot activator tube (white cap) (Sarstedt, 46.361)
- Sodium heparin tube (green cap) (Greiner, 455051)

Inco sheet
Ziploc plastic bag

b. Additional equipment:

Non-sterile gloves
2 x Baby Biome Study labels
2 x woman's hospital sticker

Timing of sample collection

Samples should be collected by the attending midwife on the labour ward as soon as possible after delivery but without causing any delay or disruption to the birth or clinical processes after birth.

Procedure

Parents may decline to give the sample but continue to provide the other samples requested.

1. Attach the woman's hospital sticker to the Baby Biome Study label.
2. Write the date and time that each blood sample was collected in the spaces provided on each labels.
3. Attach one completed Baby Biome Study label to each of the blood collection bottles.
4. Put on non-sterile gloves.
5. Place the placenta and the cord onto an inco sheet in the delivery room.
   NOTE: Do not remove the Spencer-Wells clamp from the cord before starting sample collection.
6. Take a butterfly needle from the 'cord blood collection kit'.
7. Attach the butterfly needle to the syringe, checking that the butterfly needle is well attached. Remove needle sheath.
8. Insert the butterfly needle into the **vein or artery** of the umbilical cord.
9. Attach a 20ml syringe to the butterfly tubing.
10. Fill the syringe with cord blood, avoiding drawing air bubbles though the syringe as much as possible (air bubbles damage the blood cells). Some cords may require more than one puncture site in order to collect all available cord blood.
11. Remove the syringe from the butterfly tubing.
12. Discard the butterfly needle safely into an appropriate sharps container.
13. Attach a clean butterfly onto the syringe containing the cord blood.

14. Transfer the cord blood into the appropriate blood bottles in the following order:
    a. Tempus™ Blood RNA tube (blue cap) → insert the butterfly needle into the vacutainer, transfer 3ml of blood and then discard the butterfly needle safely into an appropriate sharps container.
       NOTE: A needle is not required for transfer of samples for the remaining tubes.
    b. Serum clot activator tube (white cap) → uncap the screw cap tube and fill it with 10ml of blood and re-cap.
    c. Sodium heparin tube (green cap) (Greiner, 455051) → uncap the screw cap tube and fill it with all the remaining blood and re-cap.
15. Gently invert the full blood bottles 5-10 times to thoroughly mix the samples.
16. Discard the syringe into the clinical waste.
17. Place all the full bottles into the Ziploc bag.
18. Place the sealed Ziploc bag containing the blood samples into the “Baby Biome Study Samples” box located in the delivery suite store room.
    NOTE: Cord blood samples **must** be stored at room temperature while awaiting transfer to the laboratory.
19. Dispose of the gloves.
20. Wash your hands thoroughly.
21. Record whether a sample was given using the Baby Biome Study checklist kept in the woman’s notes. If a sample was not obtained ensure that a reason (e.g. consent declined) is provided.
22. Complete sample log on the Baby Biome Study fridge.

Cord blood sample collection: Instructions for midwives

Equipment you will need

- Non-sterile gloves
- 2 butterfly needles (size 19G) with no pre-attached vacutainer holder
- 20ml syringe
- Inco sheet
- Ziploc plastic bag
- Blood bottles:
  - Tempus™ Blood RNA Tube (blue cap)
  - Serum clot activator tube (white cap)
  - 10ml vacutainer prefilled with heparin (green cap)

Samples should be collected by the attending midwife on the labour ward as soon as possible after delivery; but without causing any delay or disruption to the birth or clinical processes after birth.

STEP 1
Print three sample stickers.
Attach these to the blood collection bottles.

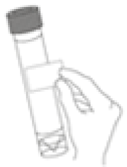

STEP 2
Put on non-sterile gloves and place the placenta and the cord onto an inco sheet.

Note: Please remember to take blood samples before removing the clamp from the cord. You may find it useful to double clamp the cord at delivery

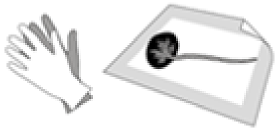

STEP 3
Take a butterfly needle and attach it to the syringe, checking that it is securely attached. Remove needle sheath.

STEP 4
Insert the butterfly needle into the vein or artery of the umbilical cord and attach a 20ml syringe to the butterfly tubing.

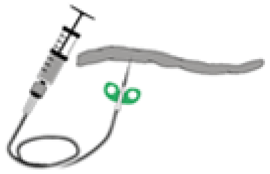

Fill the syringe with cord blood, avoiding drawing air bubbles though the syringe as much as possible. Some cords may require more than one puncture site in order to collect all available cord blood.

Note: These steps should be carried out as soon as possible because cord blood tends to clot quickly.

STEP 5
Once collection is complete remove the syringe from the butterfly tubing and discard the butterfly needle safely into an appropriate sharps container. Attach a clean butterfly onto the syringe containing the cord blood.

STEP 6
Transfer the cord blood into the appropriate blood bottles in the following order:

a. Tempus™ Blood RNA tube – insert the butterfly needle into the vacutainer, transfer 3ml of blood and then discard the butterfly needle safely into an appropriate sharps container.

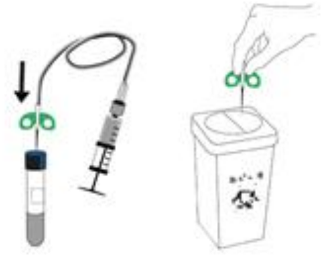

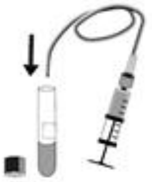

b. Serum clot activator tube – uncap the screw cap tube and fill it with 10ml of blood and re-cap.

c. 9ml vacutainer preloaded with heparin – uncap the screw cap tube and fill it with blood and re-cap.

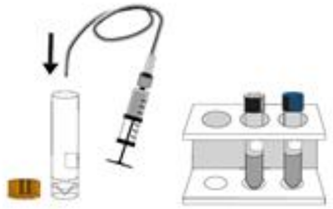

STEP 7
Gently invert each of the blood bottles 5-10 times to thoroughly mix the samples.

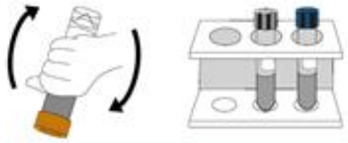

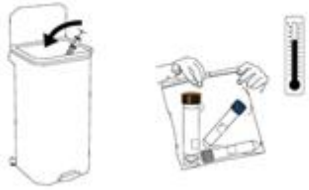

STEP 8
Discard the syringe into the clinical waste and place all the full bottles into the Ziploc bag.
Place the sealed Ziploc bag containing the blood samples into the samples box located in the labour ward sluice room.

Note: Cord blood samples must be stored at **room temperature** while awaiting transfer to the laboratory.

STEP 9
- Dispose of the gloves and wash your hands thoroughly. Record whether a sample was obtained using the midwife checklist kept in the woman's notes. If a sample was not obtained ensure that a reason (e.g. consent declined) is provided.
- Complete log book in labour ward sluice room, recording the date and time that the sample was collected.

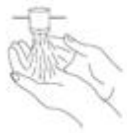

Figure 5 **Steps for cord blood collection by midwives**

2.4. Baby stool collected at home

Equipment

a. Collection kit for parents:

Sample collection instruction sheet
Stool collection tube with scoop built into the lid (BOET07.033.3107, VWR International)
Ziploc plastic bag
A prepaid, pre-labelled Royal Mail jiffy bag
Baby stool collection form

b. Additional equipment:

1 x Baby Biome Study label
2 x woman's hospital sticker

Timing of sample collection

Samples are to be collected at home by the baby's parents one week after birth and on days 4 and 21 after birth if the parents agree to provide these two additional samples.

Procedure

Explain fully to parents what the procedure involves before discharge from hospital and the reasons behind why the sample is being taken. Before issuing the collection kit, ensure that the parents remain willing to provide the sample. Parents may decline to give the sample but continue to provide the other samples requested.

Midwives should give the stool collection kit to parents before they leave the postnatal ward.

a. Instructions for midwives:

1. Before giving the kit to the woman, attach one of her hospital stickers to the Baby Biome Study label.
2. Attach the Baby Biome Study label onto the stool collection tube.
3. Attach the second hospital sticker to the designated space on the baby stool collection form inside the collection kit.
4. Give the collection kit, including the collection tube, Ziploc bag, pre-labelled Royal Mail jiffy bag, the baby stool collection form and sample collection instructions, to the parents before they leave the labour ward.
   NOTE: It is essential that parents are given the kit **before** leaving the labour ward.
5. Record whether the home stool collection kit was issued using the Baby Biome Study checklist kept in the woman's notes. If a kit was not issued ensure that a reason (e.g. consent declined) is provided.
6. **The Baby Biome Study laboratory technicians will send a text to the parents on days 4, 7 and 21 after birth, where appropriate, to remind them to collect each sample.**

b. Instructions for parents:

Read the following instructions carefully before proceeding to collect your baby's sample.

1. Lay the nappy containing stool out flat.
2. Using the scoop built into the lid of the collection tube, collect stool from the nappy and place the scooped sample into the tube.
3. Repeat step 2 up to a maximum of three times. Try to fill the spoon with stool each time.
4. Firmly screw the lid of the collection tube.
5. Write the date and time of sample collection on to the sample collection form.
6. Place the sample tube inside the Ziploc plastic bag. Seal the bag.
7. Post the sample back to the pathology laboratory as soon as possible using the prepaid pre-labelled jiffy bag.
   If the sample cannot be posted immediately, please keep the sample cool until posted, ideally by placing it in the fridge.

Stool collection instructions for babies

You have been provided with a poo (stool) collection kit that includes a collection tube, with inner lid scoop, and a Ziploc bag to put the tube in once the sample has been collected.

Please discuss with your midwife if you have any questions or concerns; phone 07958 822818

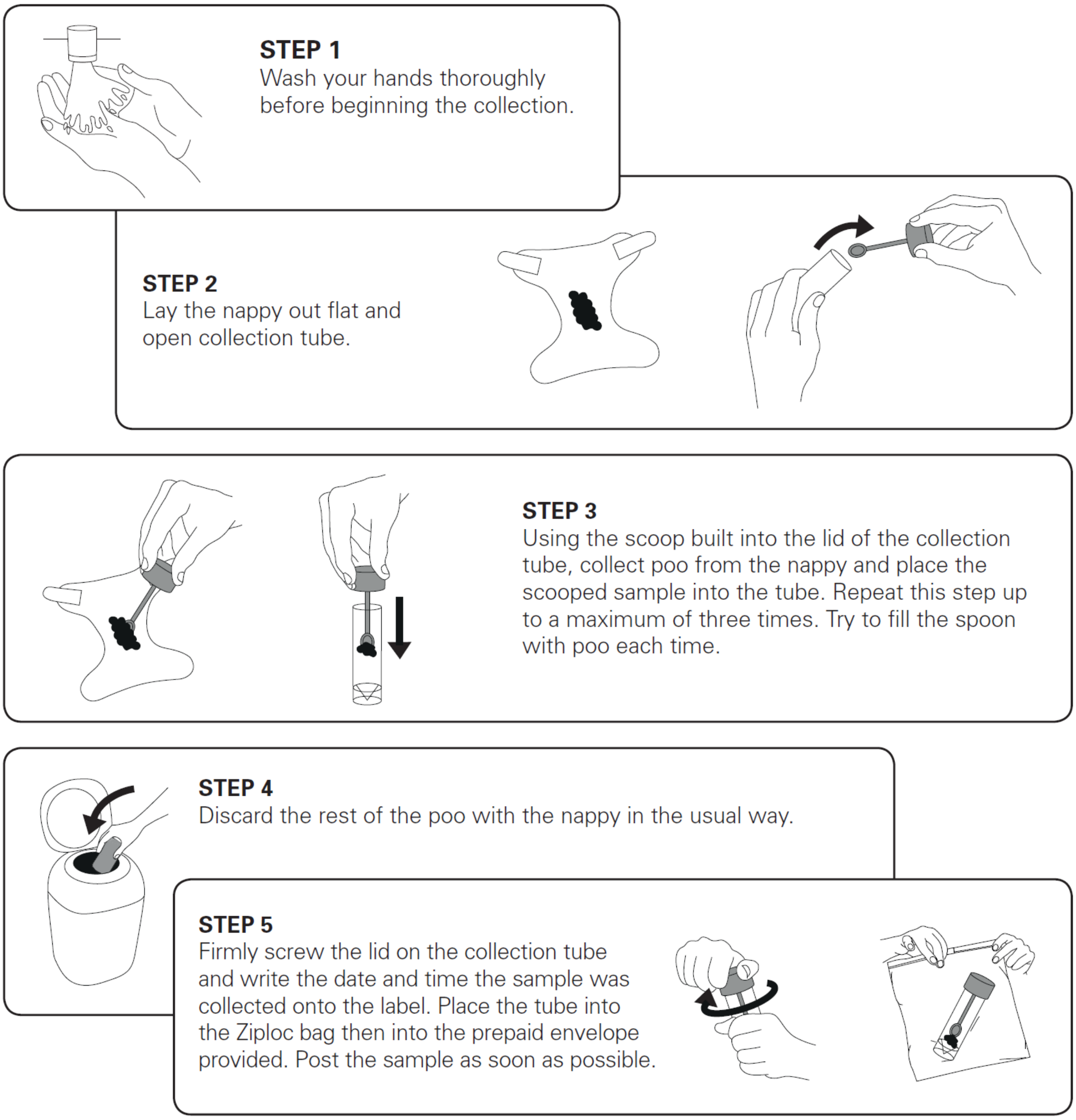

Figure 6 **Steps for home collection of baby stool**

Section C: Processing and aliquoting of samples

1. General specimen processing

1.1. Specimen handling, disposal and work bench clean up

- Use disposable latex gloves and wear a lab coat whenever handling specimens.
- Centrifuge tubes are to be capped before centrifugation and buckets are to have the lids attached. If a breakage occurs please refer to the BHRUT protocol for breakages in Appendix 1.
- Liquid waste needs to be inactivated for 24 hours in Trigene before it can be poured down the sink.
- Soak all instruments which have contacted specimens in a multi-purpose disinfectant (i.e. Trigene) then rinse thoroughly with distilled water.
- Non-disposable instruments should be thoroughly washed and autoclaved before reuse, if applicable.
- Clean the work surface with 70% ethanol before and after specimen handling.
- Never leave spillage or leaking tubes on surfaces.
- All biological/infectious wastes such as specimen containers, unused specimens and other materials (gloves, paper, wrappings, etc.), which have come into contact with biohazardous material, are to be disposed of in autoclave transparent bags (use two autoclave bags each time).
- Strippettes and all sharps including needles and scalpel blades which have come into contact with biohazardous materials should be disposed of in sharps bins.
- No mouth pipetting is permitted.

1.2. Specimen labelling

- Samples will arrive in the laboratory labelled with the woman's Baby Biome Study label and hospital sticker which together contain the following information:
    - Linear barcode for patient ID
    - Name of patient (eye readable)
    - Hospital number (eye readable)
    - Date of birth (eye readable)
    - Woman's address (UHL only)
    - Midwife name (hand written)
    - Date and time of collection (hand written)
- In the laboratory, samples will be processed and transferred to tubes for long term storage. The tubes used for storage will be pre-barcoded with a unique ID number for each aliquot.
- The aliquots will then be linked to the woman's Baby Biome Study ID, a unique and anonymised identification number for each case using a barcode scanner. The Baby Biome Study ID is generated by the Baby Biome Study technicians at the time of aliquoting.
- The following information should be recorded in the laboratory log book:
    - Baby Biome Study ID number in the following format:
        i. BHRUT: BHR00001

ii. UHL: LEI00001
iii. UCLH: UCL00001

- Date and time sample received by the laboratory.
- Specimen types collected.
- Comments: any observations or comments that may be important for the sample processing, storage, analysis and/or reporting.
- Initials of staff member making the entry.

1.3. General equipment/materials

- 1 microbiological biosafety cabinet for processing stool samples
- 1 microbiological biosafety cabinet dedicated to processing of cord blood samples (sterile environment) (optional)
- 1 centrifuge for 15ml centrifuge tubes and buckets
- 1 centrifuge for 1.5ml and 2ml Eppendorf tubes
- Racks for 1.5ml Eppendorf tubes and 15ml falcon tubes
- Vortex
- Scissors to cut the end of the P1000 tips for the initial processing of the stool samples
- Fridge (4°C)
- Freezer (-20°C, -80°C)
- Pipettes (adjustable volumes) (P200, P1000)
- Filter tips (Appleton Woods, P1000 filter tips = CD075, P200 filter tips = CD073)
- Non filter tips P1000 (Appleton Woods, CD019)
- Eppendorf DNA LoBind tubes 1.5ml PCR clean (SLS, E0030108051)
- RNase-free Microfuge Tubes (2.0 ml) (Thermo scientific, AM12425) (or any other)
- 15ml falcon tubes
- Serological pipettes (filter, plastic, sterile, individually packed) 25ml and stripettor (for DNA isolation from stool samples)
- Ethanol pure (for DNA isolation from stool samples)
- Ethanol 70% (or any other disinfectant for surfaces)
- Paper towels
- Liquid Nitrogen storage
- Nanodrop/Qubit (optional)
- FastPrep®-24 Instrument (MP Biomedicals, 116004500)
- Dry ice
- Barcoded cryogenic vials (UK Biocentre, FluidX)
- Barcode scanner for cryogenic vials
- A heatblock for 1.5ml tubes (either here or as specific equipment needed)

1.4. Handling of contaminated samples, incidents and accidents

It is inevitable that some spillages will occur during processing which may result in sample contamination or loss. In these circumstances attempts to save the sample should be made wherever possible due to their unique nature and value. However, an element of judgement

should be used to decide whether it is appropriate to save a sample. In the event of a spillage or contamination, the relevant BHRUT protocols should be followed:

- 469 – Pathology safety rules
- 483 – Chemical/biological spill
- 1524 – Control of clinical material
- 1544 – Decontamination policy
- 5543 – Disposal of waste in microbiology

All spillages which result in sample contamination and/or loss of a Baby Biome Study sample should be recorded for audit purposes using the CRM and using the Baby Biome Study auditing forms (see section 1.5 below).

1.5. Reporting of contaminated samples

Incidents such as spillages or sample contamination or accidents that occur in the laboratory involving Baby Biome Study samples should be recorded using the Baby Biome Study auditing forms (Sample Auditing Log and Incidents and Accidents Log, Appendices 1a and 1b). These forms should be kept as hard copies in the Baby Biome Study folder in the pathology laboratory.

An email notification of each incident or accident should be sent to Dr. Evi Tsaliki at UCL as and when they occur. Paper copies of auditing forms will be reviewed by Dr. Tsaliki on a monthly basis.

> **NOTE:** It is **only** necessary to fill out a BHRUT IR1 form for incidents involving Baby Biome Study samples where there is a risk to an individual's health or wellbeing. These forms **do not** need to be completed for spillages or contamination of Baby Biome Study samples.

1.6. Transfer of samples to the laboratory

All samples collected at BHRUT will be held in the Baby Biome Study fridge or a box at room temperature in the labour ward sluice room. The laboratory technician will be responsible for transferring samples to the pathology laboratory twice daily and once a day at weekends. Samples not immediately processed should be stored in the pathology laboratory at the appropriate temperature. If the labour ward fridge becomes full midwives may call the laboratory and request that the Baby Biome Study technician transfers the samples.

Samples from University Hospitals Leicester (UHL) will be sent by courier (CitySprint) every Thursday at 11am, arriving at BHRUT approximately three hours after leaving Leicester. Samples will be checked and packed by the Research Support Officer at UHL and sent with paper copies of the sample log, which will contain the following information:

1. Woman's name
2. Sample type collected
3. Date and time of collection

An electronic Excel copy of the sample log will also be sent *via* email to the Baby Biome Study nhs.net account.

Baby stool samples from all hospital sites will be returned directly to the pathology laboratory by mothers in Baby Biome Study labelled jiffy bags, by First Class post. The laboratory technicians should check the microbiology post box once each week day for returned samples that need processing.

For baby stool samples collected at UHL, the Research Support Officer will send an electronic Excel copy of the enrolment log twice per week on Monday and Friday to ensure that the laboratory technicians can send text messages to women who have agreed to provide day 4 baby stool samples. This log will contain the following information:

1. Woman's name
2. Woman's contact details (including mobile number)
3. Enrolment date
4. Woman's hospital number
5. Baby's date of birth
6. Number of baby stool kits issued

2. Sample specific processing

2.1. Vaginal swab

Specimen transport and receipt

The sample will arrive in the laboratory in a Ziploc plastic bag at ambient temperature having been stored at 4°C on the labour ward. The swab will be in a collection tube containing 1ml of Amies transport media. Once transferred, the sample should be stored in the pathology laboratory cold room (4°C) in the Baby Biome Study box until further processing.

Samples from UHL will be received weekly, on a Thursday (unless otherwise indicated), and delivered directly to the pathology laboratory by a CitySprint courier. Samples will have been transported at ambient temperature therefore must be stored at 4°C as soon as possible after receipt.

Specific equipment/materials required

Non-filter tips P1000 (Appleton Woods, CD019)
Non-sterile gloves

Processing

1. Work at the bench. Wear a lab coat and non-sterile gloves.
2. Remove the collection tube containing the swab from the Ziploc plastic bag and vortex for 10 seconds.
3. Unscrew the cap of the collection tube and, holding the top of the swab handle, press the head of the swab against the inside top of the collection tube to squeeze out any Amies media that has been absorbed by the swab.
4. Once the swab head appears to be dry, discard the swab.
5. Using a non-filter P1000 pipette split the sample into two equal aliquots and transfer into each of the cryogenic vials.
6. Scan each cryogenic vial using the barcode scanner to link the vials to the woman's Baby Biome Study ID in the CRM.

7. Store aliquots at -80°C.

Sample information to record

Time sample is processed / aliquoted
Volume / amount of each aliquot
Actual number of aliquots stored

Disposal

Please refer to BHRUT SOPs listed in section B, 1.4.

Quality control criteria

None

2.2. Maternal stool

Specimen transport and receipt

The sample will arrive in the laboratory in the Fisher specimen pot at ambient temperature having been stored at 4°C on the labour ward. Once transferred, the sample should be stored in the pathology laboratory cold room (4°C) in the Baby Biome Study box until further processing.

Samples from UHL will be received weekly, on a Thursday (unless otherwise indicated), and delivered directly to the pathology laboratory by a CitySprint courier. Samples will have been transported in a plastic "Safety Bag" (UN3373) at ambient temperature therefore must be stored at 4°C as soon as possible after receipt.

Specific equipment/materials required

FastPrep®-24 Instrument (MP Biomedicals, 116004500)
FastDNA™ SPIN Kit for Soil (MP Biomedicals, 116560200)
1 x PBS solution (Lonza, BE-17-512F)
Non-filter tips P1000 (Appleton Woods, CD019)
Filter tips P200 (Appleton Woods, CD073)
Filter tips P1000 (Appleton Woods, CD075)

Processing

Prepare all the necessary reagents, materials, and equipment at the proper temperature before starting.

1. DNA extraction

Please note that by the end of this protocol you will have prepared 4 cryogenic vials containing raw stool and 2 cryogenic vials containing extracted DNA.

NOTE: It is very important that the DNA extraction process is carried out continuously, **without breaks**, especially after loading the samples onto the spin filter.

Follow the FastPrep DNA Spin Kit for Soil as follows:

1. Work in a microbiological safety cabinet for the first part of this protocol. Wear a lab coat, non-sterile gloves and a mask.
2. Clean the work surface using 70% ethanol before starting.
3. Use 6 clean cryogenic vials to aliquot the sample:
   a. Use 4 vials for aliquoting raw stool. These vials will be stored at -80°C immediately after weighing the sample.
   b. Use 2 vials for aliquoting extracted DNA. These vials will not be needed until step 27 of this protocol.
4. Weigh the samples.
   NOTE: Before weighing your samples, the analytical balance should be tared. Taring refers to setting the scale back to read zero.
5. **FOR RAW STOOL STORAGE:** Use an empty cryogenic vial to tare the balance. With the use of non-filter P1000 tips transfer 1.5ml of sample into each of the 4 clean cryogenic vials. NB: Use the tips as sticks (instead of a spatula) and not to aspirate the samples. Weigh each vial, recording the weight in the CRM.
6. Scan each cryogenic vial using the barcode scanner to link the vials to the woman's Baby Biome Study ID in the CRM.
7. Store the 4 cryogenic vials containing the weighed raw stool sample at -80°C.
8. **FOR DNA EXTRACTION:** Use an empty Eppendorf tube to tare the balance. Transfer approximately 50mg of the sample into a clean Eppendorf tube and weigh the tube, recording the weight in the CRM.
   NOTE: For the rest of this protocol use the Eppendorf tube containing 50mg of weighed sample.
9. Dilute the sample in PBS (not provided with the kit) to a final concentration of 100mg/ml. For example, if you have 50mg of a sample, to achieve a concentration of 100mg/ml you should add 500µl of PBS.
   NOTE: This step should be carried out in a 1.5ml Eppendorf tube.
   CALCULATION: 500µl=0.5ml so 50mg/100mg/ml=0.5ml (V=m/C).
10. Add 978µl Sodium Phosphate Buffer and 122µl MT Buffer to the Lysing Matrix E Tube (all provided in the kit).
11. Thoroughly mix the diluted sample in the Eppendorf tube by vortexing. Cut a P1000 tip with the scissors. Using the cut P1000 tip, add 300µl of the diluted and vortexed sample to the LME Tube.
   NOTE: Include a positive control (300µl of a maternal sample that has been used for DNA extraction again in the past) in the first run of each kit (that is every time you open a new kit) and a negative control (300µl of PBS or sterile water) in every run of each kit (for example in every set of 12 samples). Record which samples are linked to each positive and negative control. Each positive and negative control should be stored at -20°C and once per month sent for PCR at UCL to ensure that the reagents work (clear PCR product for the positive control) and there isn't any contamination (no PCR product for the negative control).
   NOTE: The remainder of this protocol can be carried out at the bench and does not require use of the microbiological cabinet.
12. Place the LME Tube in the FastPrep Instrument (homogenizer) and run for 40secs at speed 6.
13. Centrifuge the LME tube for 10mins at 14000g.

14. Transfer the supernatant to a 1.5ml Eppendorf tube. Add 250µl of PPS (Protein Precipitation Solution) and mix by inverting the tube by hand 10 times.
15. Centrifuge the 1.5ml Eppendorf tube for 5mins at 14000g to precipitate the pellet.
16. Transfer the supernatant to a clean 15ml Falcon tube and add 1ml of Binding Matrix Solution (ensure it is well re-suspended before use; use long P1000 tips).
17. Invert tubes by hand for 2mins to allow binding of DNA. Place tube in a rack for 5mins to allow settling of silica matrix.
18. Remove 700µl of supernatant and discard it. Be careful to avoid disturbing settled Binding Matrix.
19. Re-suspend the Binding Matrix in the remaining amount of supernatant and transfer 700µl to a Spin Filter and Catch Tube.
20. Centrifuge for 1min at 14000g, discard the flow through.
21. Repeat steps 19-20 until all Binding Matrix has been transferred to the Spin Filter.
    NOTE: It essential that the samples are processed straight away, without breaks.
22. Add 500µl of SEWS-M (ethanol added) to the Spin Filter and gently re-suspend the pellet using the pipette.
23. Centrifuge for 1min at 14000g, discard the flow through and then centrifuge (without any addition of liquid) for another 2mins (rotating by 180° after 1min).
    NOTE: During centrifuging the pellet may collect on one side of the tube. This is why you need to rotate it after 1min.
24. Remove Spin Filter and place into a new Catch Tube and leave to air dry for 5mins at room temperature.
25. Add 60µl of DES (DNase/Pyrogen-Free Water) over the surface of the Binding Matrix.
26. Centrifuge for 3mins at 14000g to elute the DNA, remove the Spin Filter and discard it.
27. Perform a Nanodrop measurement of DNA concentration.
28. Split the DNA sample into two equal aliquots into the two cryogenic vials allocated in step 3b.
29. Scan each cryogenic vial using the barcode scanner to link the vials to the woman's Baby Biome Study ID in the CRM.
30. Store aliquots at -80°C.
31. Clean the work space with 70% ethanol.

Sample information to record

Time sample is processed / aliquoted
Sample volume / amount
Volume / amount of each aliquot (before DNA extraction)
Lot number of the DNA extraction kit
Code number for negative control
Actual number of aliquots stored (raw)
Actual number of aliquots stored (DNA)

Disposal

Please refer to BHRUT SOPs listed in section B, 1.4.

Quality control criteria

Nanodrop measurement of DNA concentration
PCR product for the negative control
The code of the negative control used

2.3. Cord blood – Serum

Specimen transport and receipt

A serum clot activator tube containing approximately 10ml of cord blood will be transferred to the laboratory at ambient temperature.

Samples from UHL will be received weekly, on a Thursday (unless otherwise indicated), and delivered directly to the pathology laboratory by a CitySprint courier. Serum samples from UHL will already have been isolated and transferred into a 15ml Falcon tube. Samples will have been transported at ambient temperature therefore must be stored at 4°C as soon as possible after receipt.

Specific equipment/materials required

None

Processing

Prepare all the necessary reagents, materials, and equipment at the proper temperature before starting.

1. Work in a microbiological biosafety cabinet.
2. Clean the work surface with 70% ethanol.
3. Centrifuge the serum tube at 3000g for 15min at RT.
   NOTE: For optimum serum quality and recovery, it is recommended to centrifuge samples at least 30 minutes after collection to allow for satisfactory clot retraction.
4. Transfer aliquots of serum to pre-labelled cryogenic vials. Fill each vial (working volume of 0.9ml).
   NOTE: A total of 6 aliquots of serum should be obtained from each sample.
5. Scan each cryogenic vial using the barcode scanner to link the vials to the woman's Baby Biome Study ID in the CRM.
6. Clean the work surface with 70% ethanol.
7. Store aliquots at -80°C.

Sample information to record

Time sample is processed / aliquoted
Actual number of aliquots stored
Volume / amount of each aliquot

Disposal

Please refer to BHRUT SOPs listed in section B, 1.4.

Quality control criteria

Categorize the sample based on its colour:

- Good quality (yellow)
- Slight haemolysis (pink), or
- Severe haemolysis (red)

Do not process samples if:

- Sample has been mistakenly stored in the fridge/freezer
- Volume < 2ml

2.4. Cord blood – RNA

Specimen transport and receipt

A tempus tube containing the sample will be transferred to the laboratory at ambient temperature.

Samples from UHL will be received weekly, on a Thursday (unless otherwise indicated), and delivered directly to the pathology laboratory by a CitySprint courier. Samples will have been transported at ambient temperature.

Specific equipment/materials required

None

Processing

No aliquoting is required. Label samples with a pre-barcoded label from UKBC and store at -80°C until further processing.

NOTE: If samples have been incorrectly stored in the sluice room fridge after collection, they should still be stored at -80°C until further processing.

Sample information to record

TBC

Disposal

Not applicable

Quality control criteria

None

2.5. Cord blood – stimulation tube

Specimen transport and receipt

A vacutainer containing the sample will be transferred to the laboratory at ambient temperature.

This sample type is not being collected at UHL.

Specific equipment/materials required

Heatblock for 1.5ml tubes
RNAlater

Processing

1. Within 6 hours of collection, add 1ml of heparinised cord blood into an empty 1.5ml Eppendorf tube (control sample, reference) and 1ml into a 1.5ml Eppendorf tube containing 1ug LPS (stimulated sample).
   Note: Tubes containing 1ug of LPS will be provided by Dr Evi Tsaliki ready to be used. These can be kept in the fridge for up to two weeks.
2. Incubate the two tubes containing the heparinised cord blood at 37°C for 3 hours.
3. Dispense 500ul of incubated cord blood (either control or stimulated) into clean 2ml centrifuge tubes and add 800ul RNAlater (provided with the Ribopure kit).
4. You should now have 4 tubes in total (two derived from control and two from stimulated blood).
5. Label properly and store at -80°C.

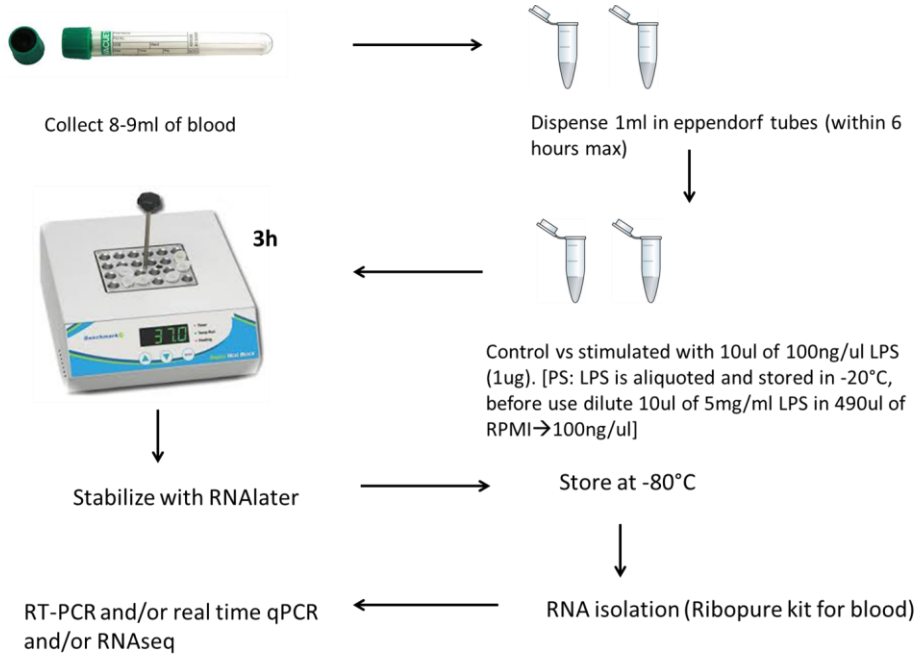

Sample information to record

TBC

Disposal

Any cord blood left will be discarded in the bins for hazardous materials.

Quality control criteria

None

2.6. Baby stool

Specimen transport and receipt

Samples will be posted to the hospital by parents in a jiffy bag labelled with the address of the pathology laboratory. Samples will arrive at the pathology laboratory at ambient temperature in the unopened jiffy bag containing the stool collection tubes inside a sealed Ziploc plastic bag. On arrival samples should be stored in the pathology laboratory cold room (4°C) until further processing.

Specific equipment/materials required

FastPrep®-24 Instrument (MP Biomedicals, 116004500)
FastDNA™ SPIN Kit for Soil (MP Biomedicals, 116560200)
1 x PBS solution (Lonza, BE-17-512F)
Non-filter tips P1000 (Appleton Woods, CD019)
Filter tips P200 (Appleton Woods, CD073)
Filter tips P1000 (Appleton Woods, CD075)

Processing

Prepare all the necessary reagents, materials, and equipment at the proper temperature before starting.

1. DNA extraction

Please note that by the end of this protocol you will have prepared 4 cryogenic vials containing raw stool and 2 cryogenic vials containing extracted DNA.

NOTE: It is very important that the DNA extraction process is carried out continuously, **without breaks**, especially after loading the samples onto the spin filter.

Follow the FastPrep DNA Spin Kit for Soil as follows:

1. Work in a microbiological safety cabinet for the first part of this protocol. Wear a lab coat, non-sterile gloves and a mask.
2. Clean the work surface using 70% ethanol before starting.
3. Use 6 clean cryogenic vials to aliquot the sample:
   a. Use 4 vials for aliquoting raw stool. These vials will be stored at -80°C immediately after weighing the sample.
   b. Use 2 vials for aliquoting extracted DNA. These vials will not be needed until step 27 of this protocol.
4. Weigh the samples.
   NOTE: Before weighing your samples, the analytical balance should be tared. Taring refers to setting the scale back to read zero.
5. **FOR RAW STOOL STORAGE:** Use an empty cryogenic vial to tare the balance. With the use of non-filter P1000 tips transfer 1.5ml of sample into each of the 4 clean cryogenic vials. NB: Use the tips as sticks (instead of a spatula) and not to aspirate the samples. Weigh each vial, recording the weight in the CRM.
6. Scan each cryogenic vial using the barcode scanner to link the vials to the woman's Baby Biome Study ID in the CRM.
7. Store the 4 cryogenic vials containing the weighed raw stool sample at -80°C.
8. **FOR DNA EXTRACTION:** Use an empty Eppendorf tube to tare the balance. Transfer approximately 50mg of the sample into a clean Eppendorf tube and weigh the tube, recording the weight in the CRM.

NOTE: For the rest of this protocol use the Eppendorf tube containing 50mg of weighed sample.

9. Dilute the sample in PBS (not provided with the kit) to a final concentration of 100mg/ml. For example, if you have 50mg of a sample, to achieve a concentration of 100mg/ml you should add 500µl of PBS.
   NOTE: This step should be carried out in a 1.5ml Eppendorf tube.
   CALCULATION: 500µl=0.5ml so 50mg/100mg/ml=0.5ml (V=m/C).
10. Add 978µl Sodium Phosphate Buffer and 122µl MT Buffer to the Lysing Matrix E Tube (all provided in the kit).
11. Thoroughly mix the diluted sample in the Eppendorf tube by vortexing. Cut a P1000 tip with the scissors. Using the cut P1000 tip, add 300µl of the diluted and vortexed sample to the LME Tube.
   NOTE: Include a positive control (300µl of a maternal sample that has been used for DNA extraction again in the past) in the first run of each kit (that is every time you open a new kit) and a negative control (300µl of PBS or sterile water) in every run of each kit (for example in every set of 12 samples). Record which samples are linked to each positive and negative control. Each positive and negative control should be stored at -20°C and once per month sent for PCR at UCL to ensure that the reagents work (clear PCR product for the positive control) and there isn't any contamination (no PCR product for the negative control).
   NOTE: The remainder of this protocol can be carried out at the bench and does not require use of the microbiological cabinet.
12. Place the LME Tube in the FastPrep Instrument (homogenizer) and run for 40secs at speed 6.
13. Centrifuge the LME tube for 10mins at 14000g.
14. Transfer the supernatant to a 1.5ml Eppendorf tube. Add 250µl of PPS (Protein Precipitation Solution) and mix by inverting the tube by hand 10 times.
15. Centrifuge the 1.5ml Eppendorf tube for 5mins at 14000g to precipitate the pellet.
16. Transfer the supernatant to a clean 15ml Falcon tube and add 1ml of Binding Matrix Solution (ensure it is well re-suspended before use; use long P1000 tips).
17. Invert tubes by hand for 2mins to allow binding of DNA. Place tube in a rack for 5mins to allow settling of silica matrix.
18. Remove 700µl of supernatant and discard it. Be careful to avoid disturbing settled Binding Matrix.
19. Re-suspend the Binding Matrix in the remaining amount of supernatant and transfer 700µl to a Spin Filter and Catch Tube.
20. Centrifuge for 1min at 14000g, discard the flow through.
21. Repeat steps 19-20 until all Binding Matrix has been transferred to the Spin Filter.
   NOTE: It essential that the samples are processed straight away, without breaks.
22. Add 500µl of SEWS-M (ethanol added) to the Spin Filter and gently re-suspend the pellet using the pipette.
23. Centrifuge for 1min at 14000g, discard the flow through and then centrifuge (without any addition of liquid) for another 2mins (rotating by 180° after 1min).
   NOTE: During centrifuging the pellet may collect on one side of the tube. This is why you need to rotate it after 1min.

24. Remove Spin Filter and place into a new Catch Tube and leave to air dry for 5mins at room temperature.
25. Add 60µl of DES (DNase/Pyrogen-Free Water) over the surface of the Binding Matrix.
26. Centrifuge for 3mins at 14000g to elute the DNA, remove the Spin Filter and discard it.
27. Perform a Nanodrop measurement of DNA concentration.
28. Split the DNA sample into two equal aliquots into the two cryogenic vials allocated in step 3b.
29. Scan each cryogenic vial using the barcode scanner to link the vials to the woman's Baby Biome Study ID in the CRM.
30. Store aliquots at -80°C.
31. Clean the work space with 70% ethanol.

Sample information to record

Time sample is processed / aliquoted
Sample volume / amount
Volume / amount of each aliquot (before DNA extraction)
Lot number of the DNA extraction kit
Code number for negative control
Actual number of aliquots stored (raw)
Actual number of aliquots stored (DNA)

Disposal

Please refer to BHRUT SOPs listed in section B, 1.4.

Quality control criteria

Nanodrop measurement of DNA concentration
PCR product for the negative control
The code of the negative control used

Section D: Shipment of samples to external sites

1. Data and sample preparation

1.1 Shipment Information Form (data preparation)

Before arranging a shipment, a 'Shipment Information Form' should be completed and sent to the Scientific Lead for approval. The form should be prepared by one of the lab technicians and checked by a second person.

NOTE: The 'Shipment Information Form' includes the following information:

- BBS ID
- Whether a valid signed consent is collected:
  Everything except the regulatory authorities' agreement (box 7) and future contact (box 8) needs to be **initialled** for consent to be valid.
- The sample types sent
- Volume, mass and/or number of aliquots sent for each sample
- Date of birth
- Date of sample collection
- Antibiotic use (in the hospital or after discharge).
- Feeding mode
- Whether other sample types were collected and stored
- Any other clinical information needed i.e. time of rupture of membranes

Please find the relevant template for each shipment in the following folders:

- Sanger Institute: Shipment Information Form_Sanger_1
- UCL Infection & Immunity (I&I): Shipment Information Form_UCL I&I_2

1.2 Sample preparation

Once the 'Shipment Information Form' has been approved by the Scientific Lead, arrange the shipment.

1. Confirm the date and time of the shipment with our partners/collaborators
2. Contact the courier to arrange pick up:
   a. For Sanger Institute: <<insert contact details>>
   b. For UCL I&I: <<insert contact details>>
3. Create a box layout to indicate the exact place of each sample in the cardboard box (if such a box is used).
4. Using the box layout, fill the cardboard box (figure) with the samples (if applicable, sealed bags or other types of racks may be used).

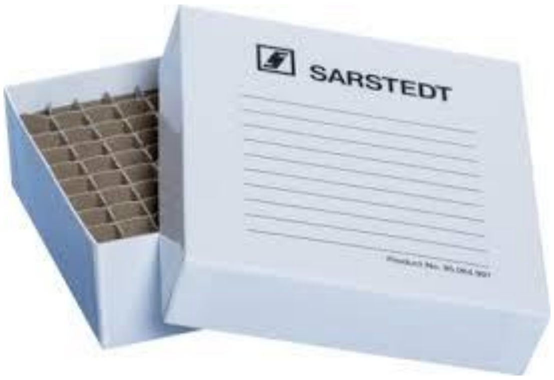

5. Place the box (or sealed bag or rack) containing the samples into a polystyrene box (figure) with dry ice ready for shipment unless otherwise stated (i.e. for samples that are not frozen).

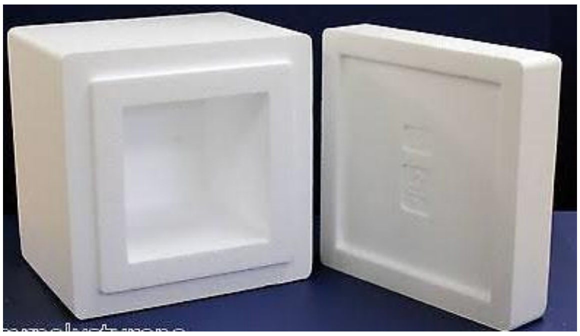

6. Send the Shipment Information Form by email to our partners/collaborators.
   NOTE: No identifiers other than the BBS ID (i.e. initials, DOB, hospital number) should be included.
   a. For Sanger Institute: <<insert contact details>>
   b. For UCL I&I: <<insert contact details>>

Section E: Appendices

1a. Sample Auditing Log

Sample auditing log

This form is used to record errors or deficiencies that occur during routine work and any corrective actions taken.

Location ____

Date ____

Report prepared by ____

Sample type:

- Cord blood (RNA) ☐
- Cord blood (serum) ☐
- Cord blood (in vivo) ☐
- Maternal stool ☐
- Baby stool ☐
- Vaginal swab ☐

Incident type:

- Spillage ☐
- Contamination ☐
- Inadequate volume ☐
- Wrong tube ☐
- Sample not labelled ☐
- Lost sample ☐
- Other (please state) ________________________________

ID of midwife who collected the sample: ________________________________

Corrective actions taken ____

____

____

____

____

____

Signed ______________________ Date ____________

Follow-up required? ☐ Yes ☐ No Date ____________________

Follow-up findings ____
____
____
____
____
____

Signed ____________________________

1b. Incidents and Accidents Log

Incidents and accidents log

This form is used to record details of any incidents or accidents.

Location ____

Date ____

Report prepared by ____

Description of event ____

____

____

____

____

____

____

Name(s) of person(s) involved ____

____

Medical treatment required? ☐ Yes ☐ No

Corrective actions taken ____

____

____

____

____

____

____

Incident reported to other departments / organisations? ☐ Yes ☐ No

Incident recorded on BHRUT system? ☐ Yes ☐ No

Signed ________________________ Date ________________
