# Supplementary material for: A pilot study to understand feasibility and acceptability of stool and cord blood sample collection for a large-scale longitudinal birth cohort
Source: BMC Pregnancy Childbirth. 2017 Dec 28;17:439. doi: 10.1186/s12884-017-1627-7 (PMC5745976; doi:10.1186/s12884-017-1627-7)
Supplement: Supplementary file 2 — Pre-birth questionnaire, pilot study questionnaire given to all women who consented to participate prior to sample collection. (PDF 368 kb) [file 12884_2017_1627_MOESM2_ESM.pdf]

Do you have any other comments or suggestions about the study?

Thank you for completing the questionnaire.
We are extremely grateful for your time and help.

Contact one of our Principal Investigators:

Mr Pat O'Brien, UCLH NHS Foundation Trust, 2nd Floor North, 250 Euston Road NW1 2PG
Professor Peter Brocklehurst, Institute for Women's Health, University College London, Medical School Building, 74 Huntley Street WC1E 6AU

Participant Identification (ID) Number:

University College London Hospitals NHS
NHS Foundation Trust

Pre-birth questionnaire
Infection and immunity pilot study

We would like you to answer some questions for us about the study materials and your feelings about being involved in the pilot study. All your answers will be kept confidential and will **not** affect your treatment or your baby's treatment in any way.

Please tick the box that best represents your views.

A Study information

How satisfied are you with the information you received about the following:

Why we want to do research into microbes (bugs, like bacteria and viruses) and the immune system?

Very Satisfied ☐ Satisfied ☐ Neither ☐ Unsatisfied ☐ Very Unsatisfied ☐

Why we need to take samples from mothers and their babies?

Very Satisfied ☐ Satisfied ☐ Neither ☐ Unsatisfied ☐ Very Unsatisfied ☐

What taking part in the pilot study would involve?

Very Satisfied ☐ Satisfied ☐ Neither ☐ Unsatisfied ☐ Very Unsatisfied ☐

B **Biological samples** *(please answer these questions even if you do **not** plan to take part in the pilot study)*

Overall, how comfortable do you feel with the idea of providing the following samples? (Note that only the samples with a star (*) next to them are being collected in this pilot study.)

Sample	Very comfortable	Comfortable	Neither	Uncomfortable	Very uncomfortable
Urine	☐	☐	☐	☐	☐
Stool (poo)*	☐	☐	☐	☐	☐
Vaginal swab	☐	☐	☐	☐	☐
Placenta (afterbirth)*	☐	☐	☐	☐	☐
Umbilical cord*	☐	☐	☐	☐	☐
Umbilical cord blood*	☐	☐	☐	☐	☐

How comfortable do you feel with the idea of the following samples being collected from your baby? (Again, only samples with a star (*) next to them are being collected in this pilot study.)

Sample	Very comfortable	Comfortable	Neither	Uncomfortable	Very uncomfortable
Saliva swab from your baby	☐	☐	☐	☐	☐
Urine from your baby's nappy	☐	☐	☐	☐	☐
Stool from your baby's nappy*	☐	☐	☐	☐	☐

How comfortable do you feel with the idea of posting a stool sample taken after birth from your baby's nappy, if we gave you a special pot and packaging, and explained carefully how to do this?

Very comfortable	Comfortable	Neither	Uncomfortable	Very uncomfortable
☐	☐	☐	☐	☐

If you feel uncomfortable or very uncomfortable about any of the above samples, please tell us why.

____

____

____

____

____

____

____

____

____

____

____

____

____

____

____

____

____

Are you planning to take part in the pilot study?

☐ Yes

☐ No

☐ Not sure
