# Supplementary material for: A pilot study to understand feasibility and acceptability of stool and cord blood sample collection for a large-scale longitudinal birth cohort
Source: BMC Pregnancy Childbirth. 2017 Dec 28;17:439. doi: 10.1186/s12884-017-1627-7 (PMC5745976; doi:10.1186/s12884-017-1627-7)
Supplement: Supplementary file 3 — Post-birth questionnaire, pilot study questionnaire given to all women who gave at least one sample. (PDF 357 kb) [file 12884_2017_1627_MOESM3_ESM.pdf]

Do you have any other comments or suggestions about the study?

Would you be willing to let us contact you again in the future? We would like to interview a small number of women who have provided samples so that we can share your experiences with other women taking part in the study. With your further permission at the time, this might involve a recorded or filmed interview. You can still decide to refuse if you change your mind when we contact you.

Yes ☐ No ☐

If yes, please provide your contact details:

Name

Phone

Email

Signature

Thank you for completing the questionnaire.
We are extremely grateful for your time and help.

Participant Identification (ID) Number:

University College London Hospitals NHS
NHS Foundation Trust

Post-birth questionnaire

Infection and immunity pilot study

We would like you to answer some questions for us about the study materials and your experience of being involved in the study. All your answers will be kept confidential and will **not** affect your treatment or your baby's treatment in any way.

You may find that you have answered some questions like this before but we are also interested in finding out if your opinions and feelings have changed from last time you gave us this feedback.

A Study information

How satisfied are you with the information you received about the following:

Why we want to do research into microbes (bugs, like bacteria and viruses) and the immune system?

Very satisfied ☐ Satisfied ☐ Neither ☐ Unsatisfied ☐ Very unsatisfied ☐

Why we need to take samples from mothers and their babies?

Very satisfied ☐ Satisfied ☐ Neither ☐ Unsatisfied ☐ Very unsatisfied ☐

What taking part in the pilot study would involve?

Very satisfied ☐ Satisfied ☐ Neither ☐ Unsatisfied ☐ Very unsatisfied ☐

B Biological samples

Overall, how comfortable did you feel about providing the following samples? If you did not provide some of the samples listed, how comfortable would you be with providing those?

Sample	Very comfortable	Comfortable	Neither	Uncomfortable	Very uncomfortable
Urine	☐	☐	☐	☐	☐
Stool (poo)	☐	☐	☐	☐	☐
Vaginal swab	☐	☐	☐	☐	☐
Placenta (afterbirth)	☐	☐	☐	☐	☐
Umbilical cord	☐	☐	☐	☐	☐
Umbilical cord blood	☐	☐	☐	☐	☐

How comfortable did you feel about the following sample being collected from your baby?

Sample	Very comfortable	Comfortable	Neither	Uncomfortable	Very uncomfortable
Stool (poo) from your baby's nappy	☐	☐	☐	☐	☐

Contact one of our Principal Investigators:

Mr Pat O'Brien, UCLH NHS Foundation Trust, 2nd Floor North, 250 Euston Road NW1 2PG
Professor Peter Brocklehurst, Institute for Women's Health, University College London, Medical School Building, 74 Huntley Street WC1E 6AU

If you feel uncomfortable or very uncomfortable about any of the above samples, please tell us why.
